# An Observational Study Suggests That Natural HAdV-36 Infection Decreases Blood Glucose Levels without Affecting Insulin Levels in Obese Young Subjects

**DOI:** 10.3390/v16060922

**Published:** 2024-06-05

**Authors:** Inés Matia-Garcia, Jorge Adalberto Ocampo-Galeana, José Francisco Muñoz-Valle, José Guadalupe Soñanez-Organis, Ramón A. González, Iris Paola Guzmán-Guzmán, Linda Anahi Marino-Ortega, Isela Parra-Rojas

**Affiliations:** 1 Obesity and Diabetes Research Laboratory, Faculty of Biological Chemistry Sciences, Autonomous University of Guerrero, Chilpancingo de los Bravo 39087, Guerrero, Mexico; 2 Research Institute in Biomedical Sciences, University Center for Health Science, University of Guadalajara, Guadalajara 44340, Jalisco, Mexico; 3 Department of Biological and Agricultural Chemistry Sciences, University of Sonora, Campus Navojoa, Navojoa 85880, Sonora, Mexico; 4 Research Center in Cellular Dynamics, Research Institute in Basic and Applied Sciences, Autonomous University of the State of Morelos, Cuernavaca 62209, Morelos, Mexico

**Keywords:** adenovirus 36, insulin resistance, obesity, glycemia

## Abstract

Human adenovirus-36 (HAdV-36) infection has been linked to obesity, low lipid levels, and improvements in blood glucose levels and insulin sensitivity in animal models and humans, although epidemiological studies remain controversial. Therefore, this study investigated the relationship between HAdV-36 seropositivity and glycemic control in youths. This observational study examined 460 youths (246 with normal weight and 214 obese subjects). All participants underwent assessments for anthropometry, blood pressure, circulating fasting levels of glucose, lipids, insulin, and anti-HAdV-36 antibodies; additionally, the homeostatic model assessment of insulin resistance (HOMA-IR) was calculated. In all, 57.17% of the subjects were HAdV-36 seropositive. Moreover, HAdV-36 seroprevalence was higher in obese subjects compared to their normal weight counterparts (59% vs. 55%). BMI (33.1 vs. 32.3 kg/m^2^, *p* = 0.03), and waist circumference (107 vs. 104 cm, *p* = 0.02), insulin levels (21 vs. 16.3 µU/mL, *p* = 0.003), and HOMA-IR (4.6 vs. 3.9, *p* = 0.02) were higher in HAdV-36-positive subjects with obesity compared to seronegative subjects. In the obese group, HAdV-36 seropositivity was associated with a reducing effect in blood glucose levels in a model adjusted for total cholesterol, triglyceride levels, age and sex (β = −10.44, *p* = 0.014). Furthermore, a statistically significant positive relationship was observed between HAdV-36 seropositivity and insulin levels in the obesity group. These findings suggest that natural HAdV-36 infection improves glycemic control but does not ameliorate hyperinsulinemia in obese subjects.

## 1. Introduction

Infectobesity is a novel concept that suggests the contribution of infectious agents to the etiology of obesity, particularly the role of viruses [1]. Adenoviruses, Borna disease viruses, canine distemper virus, and Rous-associated virus 7 have been implicated in the development of obesity [2]. Adenovirus 36 (HAdV-36) was the first human adenovirus identified as a causal agent of obesity in experimentally infected animals [3]. In fact, the causal relationship between three human adenoviruses, HAdV-36, -37, and -5, and obesity has been determined; however, only HAdV-36 has been shown to induce obesity in humans [4]. HAdV-36 is transmitted via fecal-oral, fomite, venereal, droplet, and respiratory routes in humans [1].

In animal models, experimental infection with HAdV-36 in chickens and mice resulted in increased adiposity and a decrease in blood lipid levels, particularly triglycerides and cholesterol [3]. In a rat model inoculated intraperitoneally with HAdV-36, the virus induced an increase in body weight and adiposity, mainly visceral fat, but also improved insulin sensitivity and decreased norepinephrine and corticosterone levels [5]. Similarly, two studies were conducted on non-human primates, one a longitudinal study on Rhesus monkeys with natural infection and the other on male marmosets inoculated with HAdV-36. Both experiments found that primates with positive HAdV-36 antibodies had an increase in body weight and a reduction in serum cholesterol levels [6].

Epidemiological studies in human populations have associated HAdV-36 with obesity and lower serum levels of triglycerides and cholesterol. A systematic review of 37 observational and quasi-experimental studies found a positive association between HAdV-36 seropositivity with body weight, obesity, or metabolic alterations in 31 studies. However, four studies did not find this association, and in another study, the presence of antibodies was a protective factor against obesity [7].

Some human studies have reported an association between HAdV-36 seropositivity and improved glycemic control. In a retrospective study of four cohorts that included more than 1500 subjects, individuals positive for HAdV-36 antibodies had significantly lower fasting glucose and insulin levels and lower insulin resistance, independent of sex, age, race, and adiposity, compared to HAdV-36 seronegative individuals [8].

In a longitudinal study conducted on non-diabetic Hispanic Mexican Americans, HAdV-36 seropositivity was associated with better initial glycemic control, and after 10 years, the normal weight group presented lower fasting insulin levels [9]. In a cohort of adolescents, compared with their seronegative counterparts, seropositive subjects with obesity presented lower blood glucose levels, although no relationship was observed between antibody positivity and insulin levels or insulin resistance [10].

The HAdV-36-dependent reduction in lipid levels has been demonstrated in animal models and seroepidemiological studies in humans; however, few studies have investigated the association between past HAdV-36 infection and insulin and glucose levels. Therefore, the aim of this study was to analyze the relationship between HAdV-36 seropositivity, insulin resistance, and glycemia in obese and normal weight youths.

## 2. Materials and Methods

### 2.1. Subjects and Clinical Measurements

This case-control study included 460 youths from Guerrero, Mexico, comprised of 246 normal weight individuals and 214 obese individuals. This study received approval from the Research Ethics Committee of the Autonomous University of Guerrero (CB-004/2017) and adhered to the principles outlined in the Declaration of Helsinki. Prior to inclusion, all participants received both oral and written information regarding the study and provided signed informed consent. A comprehensive clinical assessment was conducted for each participant, encompassing measurements of weight, height, body circumferences, and blood pressure. Body composition was assessed using a bioelectrical impedance analysis with a Tanita TBF-300 (Arlington Heights, IL, USA). Body circumferences were measured in duplicate to an accuracy of 0.1 cm using a Seca 201 tape measure (Hamburg, Germany). Blood pressure readings were measured twice with a baumanometer (HEM-712C, Omron Healthcare Inc., Hoffman Estates, IL, USA) to ensure accuracy. To integrate the groups, the body mass index of the subjects was considered, with a BMI ≤ 24.9 kg/m^2^ for the normal-weight group and a BMI ≥ 30 kg/m^2^ for the obesity group. Inclusion criteria were subjects without renal, hepatic, autoimmune, or thyroid disease, nor infectious diseases and without pharmacological treatment. Pregnant women or women undergoing hormonal treatment were excluded.

### 2.2. Laboratory Measurements

Participants were required to fast for 12 h overnight before blood samples were collected via venipuncture from the antecubital vein. Total cholesterol (TC), triglycerides (TG), high-density lipoprotein-cholesterol (HDL-C), and low-density lipoprotein-cholesterol (LDL-C) levels were quantified using enzymatic assays and commercially available kits (Spinreact, Barcelona, Spain) on a Mindray BS-200 clinical chemistry analyzer. Serum insulin levels were determined with an enzyme-linked immunosorbent assay (ELISA) utilizing a kit from Invitrogen, Life Technologies, CA, USA. Insulin resistance was defined based on a homeostatic model assessment of insulin resistance (HOMA-IR) with a cut-off value ≥3.2. The detection of human adenovirus 36 antibodies (AdV36-Ab) was performed in duplicate using an ELISA kit (MyBioSource, #MBS9310682, San Diego, CA, USA). The kit uses an indirect enzyme-linked immunosorbent assay to qualitatively analyze human adenovirus 36 antibody in human serum samples. The optical density of each well of the microplate was determined at a wavelength of 450 nm. The average optical density value of ≥1.0 was scored as a positive assay for HAdV-36 antibodies. The intra-assay coefficient of variation was less than 15%.

### 2.3. Statistical Analysis

Data were analyzed using STATA software (V.13.0). The comparison of characteristics between groups was carried out using Mann–Whitney U test for non-parametric data, with a Bonferroni correction test to reduce the effect of data dispersion. Multiple linear regression models were employed to analyze the associations between HAdV-36 seropositivity and metabolic measurements, and these models were adjusted for age, sex, and cholesterol and triglycerides levels. A *p*-value of <0.05 was considered statistically significant.

## 3. Results

Within the young subjects, 57.17% tested positive for antibodies. The prevalence of HAdV-36 was higher among obese individuals than in those of normal weight (59% vs. 55%). Key characteristics such as age (24 vs. 22 years, *p* = 0.02), BMI (33.1 vs. 32.3 kg/m^2^, *p* = 0.03), waist circumference (107 vs. 104 cm, *p* = 0.02), insulin levels (21 vs. 16.3 µU/mL, *p* = 0.003), and HOMA-IR (4.7 vs. 3.9, *p* = 0.02) were significantly higher in obese subjects with HAdV-36 seropositivity compared to their seronegative counterparts. However, no significant differences were observed in other anthropometric or biochemical measurements (refer to Table 1).

The impact of HAdV-36 seropositivity on clinical and biochemical measurements was assessed through linear regression analysis for both the obese and normal weight groups under study. Within the obese group, a negative effect was observed between HAdV-36 seropositivity and glucose levels (β = −9.8, *p* = 0.03), indicating that seropositivity was associated with lower glucose levels. Conversely, a positive relation was found with insulin levels (β = 4.6, *p* = 0.03), suggesting that HAdV-36 seropositive individuals in the obese group exhibited higher insulin levels. However, in the normal weight group, HAdV-36 seropositivity did not significantly influence the studied variables (refer to Table 2).

The outcomes of multiple linear regression analyses, assessing the effect of HAdV-36 seropositivity on glucose and insulin levels, are delineated in Table 3. A consistent negative relationship was observed between HAdV-36 seropositivity and glucose levels across all adjusted models analyzed in the obese group, with the magnitude of this effect being more pronounced after adjustments for total cholesterol, triglycerides, age, and sex (β = −10.44, *p* = 0.014). While a similar trend was noted in the normal weight group, the relationship reached statistical significance in only two of the analyzed models. Contrary to these results, in the obese group, HAdV-36 seropositivity was significantly positively associated with insulin levels across all models examined. This relationship was not observed in the normal weight group, where any potential effect of HAdV-36 seropositivity was minor and without statistical significance.

## 4. Discussion

To the best of our knowledge, this is the first report of the relationship between HAdV-36 seropositivity and glycemic control in Mexican youths. The HAdV-36 seroprevalence in youths was 57.17%, with a slightly higher occurrence in the obese group (59%), as opposed to the normal weight group (55%). Notably, among obese participants with positive antibodies, a favorable effect on blood glucose levels was observed, albeit without corresponding favorable changes in insulin levels and HOMA-IR. Due to the observational nature of our study, it remains beyond our scope to definitively ascertain a causal relationship between prior HAdV-36 infection and the observed decrease in glucose levels, particularly in the context of elevated insulin levels and HOMA-IR.

The HAdV-36 seroprevalence observed in this study (57.17%) is lower than the 73.9% prevalence identified in our previous study involving a Mexican child [11]. Conversely, another study focusing on obese Mexican patients with type 2 diabetes reported a seroprevalence of 41.7%, which is lower than our findings reported in this study [12]. When juxtaposed with the following data from adult populations worldwide, the seroprevalence rates in our study also appear distinct: 47.1% in Arab populations [13], 20.8% in North Americans [14], 43.3% in Italians [15], 34.3% in Koreans [16], 48.5% in Chileans [17], and 49.8% in Chinese Han [18]. The observed disparities in seroprevalence across different studies may stem from various factors, including the methodologies employed for detecting antibodies against HAdV-36, the time of evolution of viral infection, sample size, age, obesity status, and ethnicity, among others.

Few epidemiological studies have analyzed the relationship between HAdV-36 seropositivity and glycemic control. In a longitudinal study of non-diabetic Hispanic adults, seropositive non-obese subjects exhibited lower blood glucose levels at baseline, and a decade later, they also showed lower fasting insulin levels [9]. Another study highlighted a lower prevalence of HAdV-36 seropositivity among Swedish adults diagnosed with type 2 diabetes, as well as within the subgroup of women with prediabetes, compared to individuals with normal glucose tolerance. Particularly, HAdV-36-positive females with prediabetes presented with decreased HOMA-IR and lower serum insulin levels relative to seronegative counterparts [19]. Furthermore, a study conducted with adults in southern Chile found that the presence of antibodies against HAdV-36 was associated with a significant reduction in glycemia, insulinemia, and HOMA-IR among obese subjects who did not receive anti-diabetic treatment [17]. Collectively, these findings suggest that individuals with a history of HAdV-36 infection tend to exhibit lowered blood glucose levels; however, not all epidemiological studies have observed enhancements in insulin sensitivity, mirroring the outcomes of our research. Therefore, it remains to be elucidated whether improved glycemic control of HAdV-36 seropositive subjects is dependent or not of the action of insulin.

In vivo experiments have demonstrated that HAdV-36 induces beneficial changes in glucose metabolism. In studies involving male Wistar rats infected with HAdV-36, notable outcomes included increased adiposity, reduced blood lipid levels, enhanced insulin sensitivity, and a reduction in hypothalamic monoamines. Notably, low norepinephrine levels may potentially increase food intake, whereas reduced corticosterone levels might boost insulin sensitivity by promoting glucose transport in adipocytes and possibly reducing lipolytic activity [5]. Another study involving mice fed either a standard chow diet or rendered diabetic through a high-fat (HF) diet and subsequently infected with HAdV-36 revealed improvements in glycemic control as determined by the decrease in fasting glucose and insulin levels. In the HF-fed mice, findings also showed that the amelioration of hepatic steatosis and the upregulation of the Ras-PI3K signaling pathway led to increased levels of GLUT1 and GLUT4 proteins in adipose tissue and skeletal muscles. These changes likely explain the enhanced glucose uptake in these tissues [8].

In addition to the findings from animal studies, in vitro experiments utilizing 3T3-L1 preadipocytes [20], as well as diabetic and non-diabetic human skeletal muscle cells [21], have demonstrated that infected cells enhance glucose uptake. This effect is achieved through upregulation of GLUT1 and GLUT4 gene expression and protein levels, facilitated by a mechanism independent of insulin signaling, specifically through Ras-mediated activation of phosphatidyl inositol 3-kinase (PI3K). Analogous outcomes were reported in assays with adipose tissue explants and human adipose-derived stem cells, indicating that HAdV-36 uses Ras signaling rather than insulin-receptor signaling for PI3K activation [22]. In further research, distinct assays established the pivotal role of the E4orf1 protein of HAdV-36, which is what mediates its antihyperglycemic effect. This protein is encoded by the *E4orf1* gene (E4 open reading frame 1) of human adenoviruses [23]. The E4orf1 protein was identified as essential for the HAdV-36 to increase glucose uptake in 3T3-L1 cells through positive regulation of the Ras/PI3K pathway. By transfecting this protein into cells or its constitutive expression, it was determined that the E4orf1 protein binds to the Drosophila discs large protein (Dlg1) to activate total Ras, particularly the H-Ras isoform, which increases uptake almost 5-fold of glucose by the cells. Additional transfection assays in preadipocytes, adipocytes, and myoblasts confirmed that E4orf1 increases glucose uptake in all cells, and in hepatocytes, it reduces glucose output [24]. Taken together, these studies confirm the theory that HAdV-36’s E4orf1 protein promotes glucose uptake in an insulin-signaling-independent manner.

Interestingly, our study reveals a nuanced association between HAdV-36 seropositivity and metabolic parameters in young obese subjects, characterized by a negative relation with fasting glucose levels and a positive relation with insulin levels. Conversely, a similar pattern was observed in the non-obese group, albeit lacking statistical significance. These results are partly different from other studies that have reported increased insulin sensitivity and reduced glycemia in seropositive subjects [8,9,17]. Other research has documented a decrease in glycemia without accompanying changes in insulin levels among HAdV-36 positive subjects [10,12]. In contrast, some studies, including our own previous work, have not identified any association. Notably, in a prior investigation, HAdV-36 DNA was identified in 31% of adipose tissue samples from women undergoing abdominal lipectomy or liposuction. Surprisingly, the highest rate of positivity was found among women of normal weight (44%), followed by those with obesity (31%) and overweight (25%), yet no correlation was found between the presence of viral DNA and clinical and metabolic parameters [25].

Interestingly, the most important effect of the virus on glycemic control was observed in the obese group, despite the common understanding that obese subjects generally exhibit insulin resistance and hyperinsulinemia compared to non-obese subjects. This aligns with our results, as the prevalence of insulin resistance was higher in the obese group compared to the normal weight group (68.7% vs. 13%). However, within the obese group, the difference in insulin resistance between seropositive vs. seronegative subjects was not statistically significant (71% vs. 65.5%). Notably, differences in body adiposity measurements (BMI and waist circumference) was observed between seropositive and seronegative obese subjects, aligning with previous studies indicating that HAdV-36 infection improves glycemic control without reducing adiposity [5,7,8,17]. This phenomenon is attributed to the E4orf1 protein of HAdV-36, which increases glycemic control independently of the proximal insulin-signaling pathway [23,24]. Although it has also been shown that the E4orf1 protein can reduce hyperinsulinemia through an “insulin sparing action”, thereby diminishing the body’s requirement for endogenous insulin for glucose disposal [26], our study paradoxically observed a positive relationship between HAdV-36 seropositivity and insulin levels. This unexpected outcome might reflect the effective pancreatic function in obese youths. Additionally, it raises the hypothesis that seropositive individuals may experience an increase in pancreatic islet size and number, as observed in transgenic mice expressing the E4orf1 protein [27]. The duration of viral infection and viral load could also play roles in these dynamics. Nonetheless, to ascertain the relationship between natural HAdV-36 infection and systemic glycemic improvement comprehensively, a large-scale longitudinal study is required.

## 5. Conclusions

Natural HAdV-36 infection is related to improved glycemic control yet is accompanied by elevated insulin levels and is especially noticeable in obese youths.

## Figures and Tables

**Table 1:** Clinical and biochemical characteristics by group according to Ad-36 seropositivity.

	Normal Weight Group (*n* = 246)			Obesity Group (*n* = 214)		
Characteristics	HAdV-36 Negative *n* = 110 (45%)	HAdV-36 Positive *n* = 136 (55%)	*p* Value	HAdV-36 Negative *n* = 87 (41%)	HAdV-36 Positive *n* = 127 (59%)	*p* Value
Age (years) ^b^	21 (20–23)	21 (19–24)	0.38	22 (20–25)	24 (20–30)	0.02
Sex *n* (%) ^a^			0.46			0.81
Male	37 (34)	52 (38)		39 (45)	59 (46)	
Female	73 (66)	84 (62)		48 (55)	68 (54)	
Weight (kg) ^b^	54.9 (51.1–61.6)	56.3 (50.9–62.4)	0.19	85.6 (79.1–96.8)	88.1 (79.7–100.3)	0.19
Height (cm) ^b^	159 (153–167)	161 (155–168)	0.08	164 (157–169)	162 (156–171)	0.38
BMI (kg/m^2^) ^b^	21.9 (20.5–23.5)	21.9 (20.6–23.5)	0.45	32.3 (30.5–34.5)	33.1 (31.3–35.5)	0.03
Waist circumference (cm) ^b^	80 (76–84)	80 (76–84)	0.44	104 (100–108)	107 (101–111)	0.02
Hip circumference (cm) ^b^	95.3 (92–98)	95 (92–98)	0.48	114 (110–120)	115 (110–120)	0.25
Waist-hip-ratio ^b^	0.84 (0.81–0.88)	0.84 (0.80–0.88)	0.41	0.92 (0.86–0.96)	0.92 (0.89–0.96)	0.11
Body fat mass (%) ^b^	21.4 (16.7–26.9)	20.9 (16.4–26)	0.22	36.3 (32–40.2)	37.4 (33.2–42.5)	0.06
Body fat mass (kg) ^b^	12.1 (9.1–15)	11.6 (9.4–14.5)	0.30	31 (27.2–35.4)	33.1 (27.8–39.1)	0.05
SBP (mmHg) ^b^	106 (101–116)	107 (101–116)	0.48	119 (113–128)	118 (109–128)	0.25
DBP (mmHg) ^b^	68 (61–73)	69 (63–73)	0.37	73 (67–80)	74 (68–81)	0.26
Glucose (mg/dL) ^b^	83 (77–92)	84 (77–90)	0.20	91 (83–97)	89 (82–97)	0.31
Cholesterol (mg/dL) ^b^	152 (139–178)	159 (138–185)	0.18	173 (153–196)	171 (147–201)	0.47
Triglycerides (mg/dL) ^b^	84 (61–116)	87 (66–127)	0.11	137 (100–186)	139 (106–182)	0.27
LDL-C (mg/dL) ^b^	108 (93–130)	103 (85–129)	0.22	124 (88–155)	122 (99–146)	0.49
HDL-C (mg/dL) ^b^	49 (39–57)	47 (37–54)	0.06	43 (36–49)	43 (37–51)	0.23
Insulin (µU/mL) ^b^	8.0 (5.2–11.9)	9.2 (5.9–12.8)	0.23	16.3 (10.5–23.5)	21.0 (13.4–29.6)	0.003
HOMA-IR ^b^	1.69 (1.13–2.56)	1.92 (1.14–2.78)	0.35	3.9 (2.42–5.5)	4.7 (2.94–6.52)	0.02
HOMA-IR status ^a^			0.21			0.43
Non-IR (<3.2)	99 (90)	115 (85)		30 (34.5)	37 (29)	
IR (≥3.2)	11 (10)	21 (15)		57 (65.5)	90 (71)	

^a^ Data are presented as n and percentage. Chi-square test. ^b^ Data are presented as the median with the 25th to 75th percentile. Mann–Whitney U test with Bonferroni correction. BMI, body mass index; SBP, systolic blood pressure; DBP, diastolic blood pressure; LDL-C, low-density lipoprotein-cholesterol; HDL-C, high-density lipoprotein-cholesterol; HOMA-IR, homeostatic model assessment of insulin resistance. *p* value < 0.05 was considered statistically significant.

**Table 2:** Effect of HAdV-36 seropositivity on clinical and biochemical measurements in both groups.

	Normal Weight Group		Obesity Group	
Characteristics	β (95% CI)	*p* Value	β (95% CI)	*p* Value
Weight (kg)	0.31 (−1.5/2.1)	0.73	2.8 (−0.5/6.2)	0.10
Height (cm)	0.72 (−0.83/2.3)	0.36	0.14 (−1.6/1.9)	0.87
BMI (kg/m^2^)	0.02 (−0.43/0.46)	0.94	0.68 (−0.29/1.7)	0.17
Waist circumference (cm)	−0.27 (−1.7/1.2)	0.71	2.1 (−0.5/4.6)	0.11
Hip circumference (cm)	−0.1 (−1.4/1.2)	0.88	0.05 (−3.0/3.1)	0.97
Waist-hip-ratio	−0.002 (−0.02/0.01)	0.74	0.08 (−0.08/0.24)	0.35
Body fat mass (%)	−0.22 (−1.5/1.0)	0.73	1.2 (−0.1/2.5)	0.07
Body fat mass (kg)	−0.14 (−1.1/0.84)	0.78	2.1 (−0.1/4.2)	0.06
SBP (mmHg)	−0.31 (−2.9/2.3)	0.82	−1.1 (−4.3/2.2)	0.52
DBP (mmHg)	0.24 (−2.0/2.4)	0.83	0.94 (−1.8/3.7)	0.50
Glucose (mg/dL)	−2.2 (−4.7/0.35)	0.09	−9.8 (−18.4/−1.3)	0.03
Cholesterol (mg/dL)	4.5 (−4.0/13.0)	0.30	2.5 (−9.0/14.0)	0.67
Triglycerides (mg/dL)	9.7 (−2.2/21.5)	0.11	4.9 (−15.8/25.6)	0.64
LDL-C (mg/dL)	0.31 (−8.3/8.9)	0.94	1.5 (−10.4/13.4)	0.80
HDL-C (mg/dL)	−2.0 (−4.6/0.6)	0.13	0.73 (−1.7/3.2)	0.56
Insulin (µU/mL)	0.9 (−0.4/2.3)	0.18	4.6 (0.4/8.7)	0.03
HOMA-IR	0.2 (−0.15/0.5)	0.32	0.62 (−0.38/1.6)	0.22

Regression coefficient β (95% confidence interval) was adjusted by age and sex. *p* value < 0.05 was considered statistically significant. BMI, body mass index; SBP, systolic blood pressure; DBP, diastolic blood pressure; LDL-C, low-density lipoprotein-cholesterol; HDL-C, high-density lipoprotein-cholesterol; HOMA-IR, homeostatic model assessment of insulin resistance.

**Table 3:** Effect of HAdV-36 seropositivity on glucose and insulin levels in both groups.

Models	Normal Weight Group		Obesity Group	
	β (95% CI)	*p* Value	β (95% CI)	*p* Value
Effect on glucose levels				
Unadjusted	−2.17 (−4.74/0.41)	0.10	−8.32 (−16.9/0.25)	0.057
Cholesterol	−2.36 (−4.92/0.20)	0.07	−8.68 (−17.2/−0.15)	0.046
Cholesterol + Triglycerides	−2.61 (−5.16/−0.1)	0.044	−9.24 (−17.52/−0.96)	0.029
Cholesterol + Triglycerides +Age	−2.48 (−5.03/0.1)	0.057	−10.40 (−18.71/−2.1)	0.014
Cholesterol + Triglycerides + Sex	−2.65 (−5.18/−0.1)	0.041	−9.24 (−17.54/−0.94)	0.029
Cholesterol+ Triglycerides + Age + Sex	−2.51 (−5.1/0.03)	0.053	−10.44 (−18.76/−2.1)	0.014
Effect on insulin levels				
Unadjusted	0.84 (−0.54/2.22)	0.230	4.95 (0.84/9.05)	0.018
Cholesterol	0.82 (−0.57/2.20)	0.246	4.85 (0.75/8.96)	0.021
Cholesterol + Triglycerides	0.61 (−0.74/1.96)	0.373	4.63 (0.60/8.67)	0.024
Cholesterol + Triglycerides +Age	0.62 (−0.74/1.98)	0.372	4.35 (0.28/8.42)	0.036
Cholesterol + Triglycerides + Sex	0.65 (−0.68/1.97)	0.337	4.64 (0.60/8.68)	0.025
Cholesterol+ Triglycerides + Age + Sex	0.65 (−0.68/1.99)	0.335	4.37 (0.28/8.45)	0.036

Regression coefficient β (95% confidence Interval). *p* value < 0.05 was considered statistically significant.

## Data Availability

The data analyzed in this study are available from the corresponding author upon reasonable request.
